# Medical News and Miscellany

**Published:** 1899-08

**Authors:** 


### Medical News and Miscellany.

**See** the advertisement of the "Cotton Belt Route" in this issue.

---

**Dr. S. E. Hudson,** of Austin, has been appointed a member of the Board of Managers of the State Insane Asylum at Austin.

---

**We publish herewith** Dr. B. M. Worsham's report to the Governor, on the subject of an epileptic asylum, or colony for epileptics in Texas.

**The** American Electro-Therapeutic Association will hold its ninth annual meeting at Washington, D. C., September 19, 20, 21, 1899. President, Dr. F. B. Bishop.

---

**Dr. Benj. Frenkel,** of Texas, surgeon U. S. Navy, has been transferred from Norfolk, Va., to San Juan, Porto Rico, and is on duty in the U. S. Naval Station Hospital at that place.

---

**The** venerable Doctor T. D. Wooten, President of the Board of Regents of the University of Texas, has resigned that position on account of failing health and has gone to Canada to recuperate.

---

**Dr. T. J. Turpin,** long time State quarantine officer at Laredo, Texas, has been appointed sanitary inspector in the U. S. Marine Hospital Service with the rank of assistant surgeon, and assigned to duty in the city of Mexico.

---

**The Annual Announcement** of the Medical Department of the University of Tennessee will be found in this issue. It was received too late for July number. Professor Eve will be pleased to send catalogue and all desired information on request.

---

**The New Orleans Polyclinic,** thirteenth annual session, opens November 20, 1899; closes May 10, 1900. Every inducement in clinical facilities for those attending. The specialties are fully taught. Further information, New Orleans Polyclinic, New Orleans, La.

---

**University of Nashville,** Medical Department.—Of 21 graduates of this school, who were applicants before the examining board of Tennessee, twenty passed; of five applicants, before the Mississippi board, four passed; and of three, before the North Carolina board, none were rejected. (From *Medical and Surgical Bulletin*, *Nashville*, *Tenn.*—August, '99.)

---

**Prof. A. C. Bernays,** of St. Louis, Mo., has resigned his chair in the Marion Sims College of Medicine, and will devote three and one-half hours each day in the future towards teaching practical surgery to private classes of graduates in medicine. Details and prospectus can be had by addressing his manager, Dr. Frank M. Floyd, 612 Union Trust Building, St. Louis, Mo.

**Change of Address.**—Dr. James Bartlett from Brenham to San Antonio. Dr. A. A. Gregory from Cleburne to Savoy. Dr. F. B. Kennedy from Peters to Dublin. Dr. J. H. Mincey from Van Alstyne to Howe. Dr. M. L. Martin from Galveston to Denton. Dr. L. B. Tenney from Crockett to Archer City. Dr. A. J. James from Caldwell to Cleburne. Dr. R. T. Blount from San San Antonio to Fairfield. Dr. C. J. Forbes from Leakey to Sabinal. Dr. Andrew Sibley from Palestine to Wortham.

---

**In this issue** we give space to an article from Dr. Herbert H. Shappard, D.D.S., who was a member of the first Texas regiment, in the late war with Spain. The doctor is a graduate of the University of Pennsylvania, and at our earnest solicition, kindly consented to give his experience as a dentist in the army. Also his humble opinion as to the advisability of adding a dental department to the surgical corps of the regular army. As this subject is now being so universally discussed in the leading dental journals, we presume the doctor's article will be of value with the advocates of this important measure.—(Ed. Dental Department.)

---

### The San Antonio Fair.

An interesting feature of the great San Antonio International Fair, to be held in the city of San Antonio, October 28, to November 8, inclusive, will be the live stock exhibit. The management of the Association states that stall room has already been engaged for upwards of 3000 head of improved cattle, horses, sheep, swine and goats, and before the gates of the Association are opened to the visitors it is reasonably certain that 5000 head of improved live stock will be on the grounds. It is already an assured fact that the live stock show at the San Antonio International Fair will be the largest ever held in Texas or the South.

Exhibitors in this department will be there from every section in Texas, and from most of the live stock centers of the North and West. San Antonio is known everywhere as being the center of the greatest live stock breeding ground in the world, and the management of this Fair has offered liberal inducements for exhibitors in this line.

Another interesting feature of this Fair will be the large number of exhibits from the farms, orchards and gardens of the State. Truck farmers of the great coast country of Texas have organized and will make a splendid exhibit, as also will the fruit growers of that and other sections. Besides, there will be numberless individual and county exhibits of farm products, and to these will be added the large exhibits to be made by the San Antonio & Aransas Pass and International & Great Northern Railroads.

All the space has already been taken in the Machinery, Implement and Exposition Halls, and visitors to the Fair will see as fine a line of exhibits from mills and factories as was ever shown at any fair south of St. Louis. Great interest is being manifested in the Ladies' and Art departments of the Fair, and exhibits in these departments will be far above the average.

The management has just issued the race program, providing for one harness and four running races each day, and the premiums are large enough to bring to this meeting the greatest number of the fastest horses ever gathered at one point in Texas. Other attractions will be numerous and varied enough to suit every taste.

---

### Ten Prize Hygienic Rules.

The following maxims won a prize offered in 1897 by the Parisian publishers, Hachette & Company, for the ten most effectual rules for the preservation of mental and bodily health. The author, Dr. Decornet, of Ferté-sur-Aube, won over five hundred competitors. The rules, as translated in *The Lancet,* run thus: 1. General Hygiene: Rise early, go to bed early, and in the meantime keep yourself occupied. 2. Respiratory Hygiene: Water and bread sustain life, but pure air and sunlight are indispensable for health. 3. Gastro-intestinal Hygiene: Frugality and sobriety are the best elixir for a long life. 4. Epidermal Hygiene: Cleanliness preserves from rust; the best-kept machines last longest. 5. Sleep Hygiene: A sufficiency of rest repairs and strengthens; too much rest weakens and makes soft. 6. Clothes Hygiene: He is well clothed who keeps his body sufficiently warm, safeguarding it from all abrupt changes of temperature while at the same time maintaining perfect freedom of motion. 7. House Hygiene: A house that is clean and cheerful makes a happy home. 8. Moral Hygiene: The mind reposes and resumes its edge by means of relaxation and amusement, but excess opens the door to the passions and these attract the vices. 9. Intellectual Hygiene: Gaity conduces to love of life and love of life is the half of health; on the other hand, sadness and gloom help on old age. 10. Professional Hygiene: Is it your brain that feeds you? Don't allow your arms and your legs to become ankylosed. Dig for a livelihood, but don't omit to burnish your intellect and elevate your thoughts. —*Literary Digest.*

---

### College of Physicians of Philadelphia.

THE WILLIAM F. JENKS MEMORIAL PRIZE.—The Fifth Triennial Prize of Five Hundred Dollars, under the Deed of Trust of Mrs. William F. Jenks, will be awarded to the author of the best essay on "The Various Manifestations of Lithæmia in Infancy and Childhood, with the Etiology and Treatment."

The conditions annexed by the founder of this prize are, that the "prize or award must always be for some subject connected with Obstetrics, or the Diseases of Women, or the Diseases of Children;" and that "the Trustees, under this deed for the time being, can, in their discretion, publish the successful essay, or any paper written upon any subject for which they may offer a reward, provided the income in their hands may, in their judgment, be sufficient for that purpose, and the essay or paper be considered by them worthy of publication. If published, the distribution of said essay shall be entirely under the control of said Trustees. In case they do not publish the said essay or paper, it shall be the property of the College of Physicians of Philadelphia."

The prize is open for competition to the whole world, but the essay must be the production of a single person.

The essay, which must be written in the English language, or if in a foreign language, accompanied by an English translation, must be sent to the College of Physicians of Philadelphia, Pennsylvania, U. S. A., before January 1, 1901, addressed to Richard C. Norris, M. D., Chairman of the William F. Jenks Prize Committee.

Each essay must be typewritten, distinguished by a motto, and accompanied by a sealed envelope bearing the same motto and containing the name and address of the writer. No envelope will be opened except that which accompanies the successful essay.

The committee will return the unsuccessful essays if reclaimed by their respective writers, or their agents, within one year.

The committee reserves the right not to make an award if no essay submitted is considered worthy of the prize.

JAMES V. INGHAM, M. D.,

June 15, 1899. Secretary of the Trustees.

